# P-1244. PK/PD Target Attainment of Beta-lactams in Patients With Versus Without Obesity

**DOI:** 10.1093/ofid/ofaf695.1436

**Published:** 2026-01-11

**Authors:** Venugopalan Veena, Logan Brock, Krysten McNaught, Nicole Maranchick, Simone Marini, Nicholas Brito, Sophia Garcia, Lemay Gonzalez, Charles Peloquin, Kathryn DeSear

**Affiliations:** University of Florida, College of Pharmacy, Gainesville, Florida; UF Health Shands Hospital, Gainesville, Florida; UF Health Shands Hospital, Gainesville, Florida; University of Florida College of Pharmacy, Gainesville, Florida; University of Florida, Gainesville, Florida; University of Florida College of Pharmacy, Gainesville, Florida; University of Florida College of Pharmacy, Gainesville, Florida; University of Florida College of Pharmacy, Gainesville, Florida; University of Florida College of Pharmacy, Gainesville, Florida; UF health Shands Hospital, Gainesville, Florida

## Abstract

**Background:**

Previous studies have shown that patients with obesity are at risk for subtherapeutic beta-lactam concentrations, owing to the pharmacokinetic (PK) changes in factors such as volume of distribution and clearance. Beta-lactam therapeutic drug monitoring (TDM) has been utilized as a strategy to reduce PK variability and individualize dosing regimens, although data exploring the use of TDM in this population are limited. This study sought to evaluate the role of TDM in patients with obesity and its impact on pharmacokinetic/pharmacodynamic (PK/PD) target attainment of beta-lactams.

**Methods:**

This single-center, retrospective, matched cohort study included adult patients with obesity (OB) and patients without (non-OB) who had routine TDM performed for cefepime (FEP), piperacillin-tazobactam (PTZ), or meropenem (MEM). Underweight patients were excluded. Patients were matched by drug, ICU status, renal function, and early (≤ 72 hours) or late TDM ( > 72 hours). The primary outcome compared PK/PD target attainment in OB vs non-OB. This study assessed two PK/PD targets (100% *f*T≥ MIC and 100% *f*T≥ 4xMIC). Secondary outcomes included treatment failure, length of stay, in-hospital mortality, adverse events, and 30-day readmission.

**Results:**

Of 199 matched pairs of patients, 298 (75%) patients received FEP, 48 (12%) received PTZ, and 52 (13%) received MEM. There was no difference in attainment of 100% *f*T≥ MIC between OB vs non-OB at 84% (n=168) and 85% (n=170), respectively. Target attainments were similar, yet much lower for 100% *f*T≥ 4xMIC in OB vs non-OB at 53% (n=106) and 54% (n=108) respectively. OB and non-OB receiving PTZ had the lowest overall rates of attainment of 100% *f*T≥ MIC (69%) and 100% *f*T≥ 4xMIC (29%). More OB experienced any adverse effect compared to non-OB at 48 (24%) and 30 (15%), respectively (p = 0.02). Specifically, rates of nephrotoxicity were greater in OB vs non-OB (15% vs. 6%, p< 0.01). There were no differences in any other secondary outcomes.

**Conclusion:**

PK/PD target attainment was comparable between OB and non-OB in this study, but lowest with PTZ, highlighting the need for targeted TDM in these patients. OB experienced significantly higher rates of adverse effects, including nephrotoxicity, emphasizing the importance of TDM in improving patient safety.

**Disclosures:**

Venugopalan Veena, PharmD, Merck: Grant/Research Support Kathryn DeSear, PharmD, Abbvie: Advisor/Consultant|Biomerieux: Advisor/Consultant|Cormedix: Speaking|GSK: Advisor/Consultant|Shionogi: Speaking

